# Influence of Er:YAG and ND:YAG laser irradiation and fluoride application on surface roughness and dentin surface wear after erosive challenge - An in vitro study

**DOI:** 10.4317/jced.60945

**Published:** 2024-03-01

**Authors:** Natyelle-Fernanda-Silva-Bellocchio Corrêa, Regina-Guenka-Palma Dibb, Vinicius-Rangel Geraldo-Martins, Isabela-Ribeiro Madalena, Juliana-Jendiroba Faraoni, Maria-Angelica-Hueb-de Menezes Oliveira, Denise-Tornavoi de Castro, Cesar-Penazzo Lepri

**Affiliations:** 1 Department of Biomaterials, University of Uberaba, Uberaba, MG, Brazil; 2 Department of Restorative Dentistry, School of Dentistry of Ribeirão Preto, University of São Paulo, Ribeirão Preto, SP, Brazil; 3 School of Dentistry, Presidente Tancredo de Almeida Neves University Center, São João del Rei, MG, Brazil

## Abstract

**Background:**

To evaluate the effectiveness of Er:YAG and Nd:YAG laser on dentin hypersensitivity prevention, associated or not to acidulated phosphate fluoride (APF) after erosive challenge.

**Material and Methods:**

104 specimens were obtained from bovine dentine and divided into groups (n=13): G1: Er:YAG; G2: Er:YAG followed by application of APF; G3: application of APF followed by Er:YAG, simultaneously; G4: Nd:YAG; G5: Nd:YAG followed by application of APF; G6: application of APF followed by Nd:YAG, simultaneously; G7:application of APF; G8: untreated. The parameters for Er:YAG were:10s, distance of 4mm, water cooling flow of 2mL/min, 2Hz, 3.92J/cm2. For the Nd:YAG: 10s, distance of 1mm, without cooling, 10Hz, 70.7J/cm2. The erosive drink was a cola at 4°C, 3×/day for 1 minute, for 5 days. Roughness and wear analysis were done in confocal laser microscope. Data were statistically analyzed (α=0.05).

**Results:**

As regards roughness, there was no statistically difference among the groups. The groups irradiated with Er:YAG had a volume loss lower. G6 showed higher values than the groups irradiated with Er:YAG and lower than the other groups. The other groups irradiated with Nd:YAG showed similar wear results to the control.

**Conclusions:**

The Er:YAG laser showed the lowest volume loss from wear analysis, suggesting the increased the acid resistance of dentin.

** Key words:**Dentine sensitivity, Lasers, Sodium fluoride.

## Introduction

The dentin hypersensitivity (DH) or hyperalgesia is understood to be a sharp pain, short, manifesting itself uncomfortably to the patient. This pain occurs as a result of exposed dentine in response to chemical, thermal, tactile or osmotic stimulus, which cannot be explained as arising from any other dental defect or disease (1). It occurs due to the presence of open dentinal tubules on an exposed dentin surface (2). Enamel and cementum loss causes dentine exposure to the oral environment (2). This loss is derived from several factors, such as sub-gingival scaling, dental crowding, or the combination of two or more factors. The combination of these factors, such as abrasion, abfraction and acid erosion also cause DH and acid erosion can arise due to extrinsic factors (acidic foods and drinks such as citrus fruits, coffee, soft drinks, wine and other alcoholic drinks) and intrinsic, caused by eating disorders and gastroesophageal disorders (anorexia, xerostomia, bulimia and acid reflux), and even the force applied during dental hygiene can be an aggravating factor of erosion (3-5).

 The most accepted theory to explain the pain transmission mechanism is the hydrodynamic theory, proposed by Brännström (6). Under this theory, exposure of dentinal tubules to the oral environment would allow the movement of dentinal fluid, thereby stimulating the nerve fibers, thus causing the pain sensation (6,7). Several methods are available for the treatment of dentin hypersensitivity, all with the same purpose: seal the dentinal tubules (8-10). Among these methods, it can be cited the use of fluoride varnishes, potassium oxalate, self-etching adhesive system, special toothpastes. Another method also used to treat tooth sensitivity is iontophoresis (11). Fluoride compounds are the most used for the reduction of dentin hypersensitivity (12). These desensitizing treatments should be used systematically, beginning with prevention and treatments performed at home with the use of fluoride dental toothpaste and complemented by dentists, with their supervision with the procedures performed at dental office (13).

The fluoride topical application prevents the dissolution of the dental substrate (14,15), consequently increasing the acid resistance of enamel, but its mechanism will depend on its ability to interfere with the demineralization and remineralization process. Another way to treat dentinal hypersensitivity may be obtained by using lasers. Currently, the laser therapy is used, with or without fluoride, with satisfactory results (16). The first laser was discovered in 1960, creating the first solid-state laser and using ruby as the medium. This laser is situated in the visible range of the electromagnetic spectrum. From the experiments carried out with the ruby laser, other lasers have been developed and used in the treatment of dentinal hypersensitivity, such as CO2, diode (GaAlAs), He-Ne, Nd:YAG, Er:YAG and Er,Cr:YSGG (17-19).

Due to the variety methods and types of lasers, it was not possible to propose a definite method for treating DH. This way, it would be interesting to obtain safe and ideal parameters using high power lasers, to get morphological changes in dental tissues, such as sealing and occlusion of dentinal tubules by melting and recrystallization of dentin. The aim of the present study was to analyse the effects of Er:YAG and Nd:YAG laser irradiation, associated or not with 1.23% sodium fluoride (NaF) application on dentin hypersensitivity prevention, after erosive challenge, assessed by surface roughness and wear analysis (confocal laser microscopy).

## Material and Methods

-Preparation of the Samples

Fifty-two bovine incisive teeth were collected and immediately stored in distilled water. The teeth that had microcracks, stains due hypoplasia or wear were discarded. After cleansing and root planning using a curette until the dentin exposition, the teeth were stored in distilled water under refrigeration at 4°C. The crowns were separated from the roots at the cement-enamel junction using a section machine (Iso Met® 1000, BUEHLER-Lake Bluff, IL 60044/USA) with a water-cooled diamond disk (Isomet; 10.2cm×0.3mm, arbour size 1/2 in., series 15HC diamond; Buehler, Lake Bluff, IL, the USA) in low speed.

**Figure 1:**
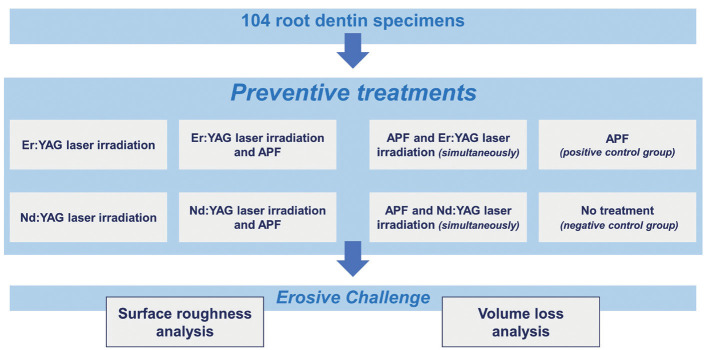
Illustrative flowchart demonstrating the random division of specimens according to the treatments performed.

Then, the roots were sectioned and divided in half to obtain 104 fragments of 4.25×4.25×3.00 mm. The specimens were delineated and polished under water cooling and sandpaper (granulation #600 and #1200). After polishing, all fragments were coated with two layers of nail varnish and wax (reference area), leaving half of the dentin surface without protection (9mm2) to apply the preventive treatments and induce erosive challenge. Afterwards, the specimens were randomly divided into eight groups according to the treatments performed (Fig. 1).

-Experimental Groups

 One hundred and four root dentin samples were randomly divided into 8 groups (n=13). The sample size was calculated considering a significance level of 5% and test power of 85%. In each sample, the delimitated area was treated according to Table 1.

**Table 1:**
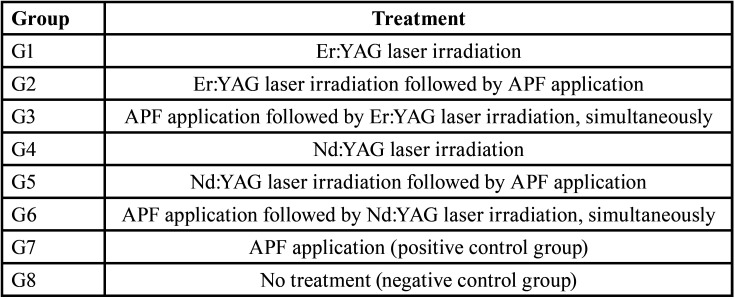
Treatment used in the different groups.

G1 was only irradiated with Er:YAG laser; G4 received only Nd:YAG laser. In G2 and G5, the NaF (1.23% fluoride gel - DFL Industria e Comercio SA - RJ/Brazil) was applied after irradiation for 4 minutes. The samples of the G3 and G6 received NaF for 1 minute, simultaneously irradiated (10 seconds) and NaF was left in the specimen until completing 4 minutes. In G7, a NaF gel was applied on the samples for 4 minutes (positive control group). For all groups that received NaF, the excess gel was removed with gauze immediately after completing the fourth minute and then the specimens were stored in distilled water at 37°C until the next step of the experiment. Finally, G8 received no treatment (negative control group).

To ensure consistent spot size with the hand irradiation, an endodontic file was fixed on the handpiece, and kept a determined distance from the surface during the irradiation procedures. The laser parameters used for laser irradiation in each group are shown in Table 2. The handpiece was positioned perpendicularly to the root dentin surface, and the samples were irradiated once in each direction, moving the handpiece slowly horizontally and vertically, to promote homogeneous irradiation and to cover the entire sample area. The irradiation was performed by hand (simulating a clinical situation) and scanning the dentin surface for 10 seconds. The output power was measured with a power meter (TM- 744D, Tenmars Electronics Co., Taipei, Taiwan). At the end of these treatments, all samples were kept in distilled water at 37°C until the next step. Afterwards, the samples of all groups were submitted to an erosive challenge.

**Table 2:**
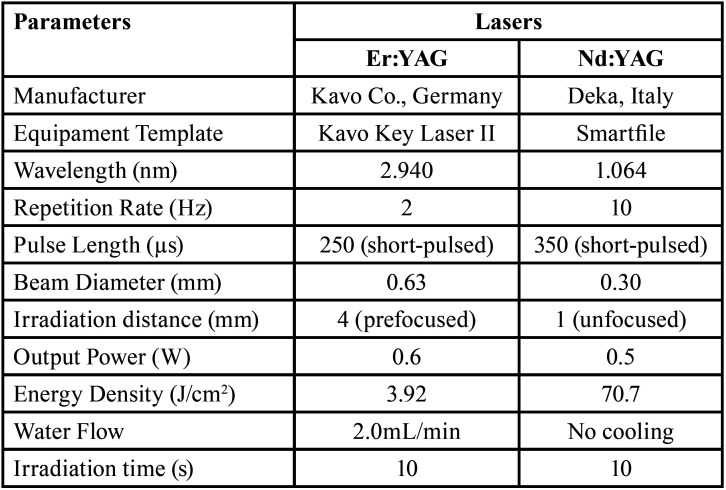
Lasers parameters of the experimental group.

-Erosive Challenge

For the erosive challenge, samples were submitted to daily immersion in 50 mL of Coca-Cola at 4oC (pH=2.42), under stirring, for one minute, three times a day. This cycle was carried out for 5 days. The specimens were stored in distilled water between the cycles. At the end of each day, these also remained in distilled water, which was daily changed.

-Surface roughness measurement and Wear analysis

 The specimens were washed with distilled water and dried with paper tissue. The wax and nail varnish were carefully removed, exposing the control area. The surface roughness and dentin wear were evaluated with a laser confocal microscope (LEXT-Olympus) connected to a computer with specific software (OLS4000).

 As regards surface roughness, each specimen was measured seven times in each area (reference or treated). This variable was evaluated in Ra parameter, measured in micrometers (ISO 25178).

The wear measurements of the treated/eroded surface were performed in relation to the untreated area (reference area). After profile determination, the wear measurement was calculated in volume (µm3), considering the medium line of the graphic (referring to the protected area = reference area) and the erosion line (treated/eroded area). Each specimen was measured in a central area of 1mm2. Finally, we considered the percentage of lost volume, comparing the treated area to the reference area.

-Statistical Analysis

For the surface roughness analysis, firstly, the assumptions of equality of variances (modified Levene equal-variance test) and the normality of the error distributions (Shapiro-Wilk test) were checked for the response variables tested. Since the assumptions were satisfied, the ANOVA test (α=5%) was applied using SPSS Statistics Version 17.0 software (Chicago: SPSS Inc.). For wear analysis, data were submitted to non-parametric test of Kruskal-Wallis followed by Dunn test, both with α=5%.

## Results

The results, expressed in Ra (µm), are described in Table 3. There was no statistically significant difference among all groups (*p*>0.05).

**Table 3:**
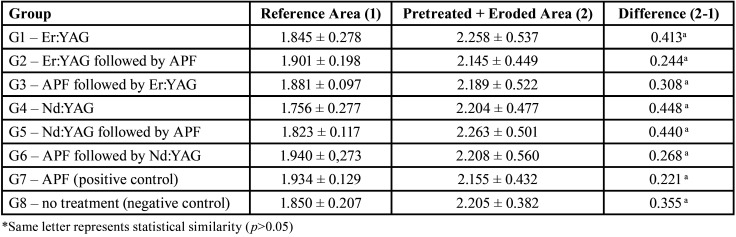
Means (µm) ± standard deviations of the surface roughness of the dentin surface after different preventive pretreatments followed by erosive challenge.

The groups irradiated with Er:YAG laser had a volume loss significantly lower when compared to other groups (*p*<0.05). G6 group (NaF application followed by Nd:YAG laser irradiation, simultaneously) showed higher values than the groups irradiated with Er:YAG and lower values than the other groups. The other groups irradiated with Nd:YAG laser showed similar wear results to the control groups (*p*>0.05). The percentages of lost volume are shown in Table 4.

**Table 4:**
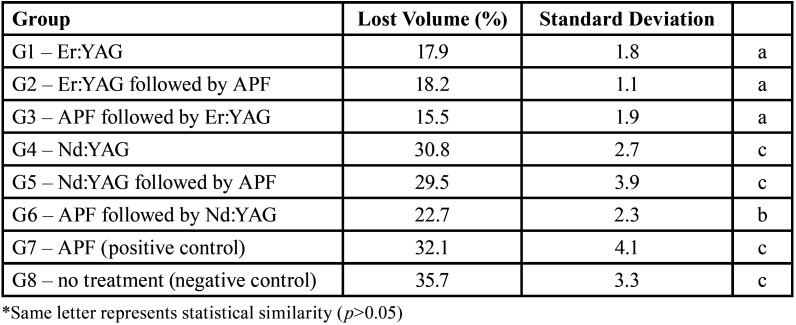
Lost volume (%) and standard deviations of the wear of the dentin surface after different preventive pretreatments followed by erosive challenge, comparing the treated area to the reference area.

## Discussion

The use of laser therapy for dentin hypersensitivity prevention has been shown to be a promising method. Our study confirmed this hypothesis.

Although there exists evidence on the effects of fluoride on dental tissue, it is also known that such methods have limited actions in an acid environment (20,21). Fluoride application leads to the formation of a calcium fluoride-like compound that is more unsTable and easily dissolved by most acidic beverages and acids from the cariogenic challenge. Thus, new technologies, including laser therapy, have been developed to allow the enamel to obtain greater resistance to acid attack (22).

Depending on the irradiation parameters, the action of high-power lasers, such as the lasers used in this study (Er:YAG and Nd:YAG), can cause fusion through the melting of the hydroxyapatite and its resolidification after cooling. This allows the formation of hydroxyapatite crystals larger than os crystals observed in the initial structure, obliterating the dentinal tubules and consequently reducing HD (23). This reduction in HD was concluded in a systematic review and meta-analysis and it was verified that it occurs even after 3 months of treatment (24). Thus, laser therapy is a choice in controlling the symptoms of painful sensitivity, although there is no defined treatment protocol. This probably occurs due to different methods of assessing pain sensitivity and the fact that the “pain” factor is subjective.

The parameters of the Er:YAG laser used to treat HD, according to Asnaashari and Moeini (25) are 1W and 10-12 Hz, with irradiation duration of less than 60 seconds, in order to prevent damage to dental surface and soft tissues. According to Aranha *et al*. (26) the Er:YAG laser is highly effective in reducing the diameter of dentinal tubules under specific conditions, with partial obliteration of the tubules. In the present study, Er:YAG and Nd:YAG lasers with sub-ablative parameters were used to obtain an adequate energy density for the prevention of dental demineralization, without damaging the surface through the ablative process. We proposed to study surface roughness because the presence of irregularities can lead to bacterial biofilm retention and gingival irritation, increasing the risk of caries and periodontal inflammation (27).

Dilber *et al*. (28) used three types of lasers: Er:YAG, Nd:YAG and KTP. They concluded that irradiation with these lasers did not affect the structure and the composition of the dentin surface. The average percentage of minerals weight, such as Ca, K, Mg, Na and *P* were not affected. Previously, in other research with Er:YAG and Nd:YAG lasers, Rohanizadeh *et al*. (28), they noted that the proportion of minerals Ca and *P* was decreased in Er:YAG irradiated tissue, and increased in the Nd:YAG irradiated tissue. This might be explained by the Nd:YAG action mechanism: the hydroxyapatite crystals melt in the presence of energy, immediately occluding the tubules. The Nd:YAG laser was effective only when it was previously performed the application of fluoride. This finding is different to that found by Raucci-Neto *et al*. (30), probably because the substrate evaluated in that study was the enamel, which has significant differences from the dentin studied in this study.

The findings in the present study suggest that the laser irradiation with both devices are effective when the roughness parameter was analyzed, however, more studies are needed to assess whether there is change in the percentage of dentin minerals. Lastly, the Er:YAG laser has been shown to be safe in dental irradiation, since it promoted accepTable temperature increases (31). Furthermore, it also presented in the present study the advantage of significantly reducing the mineral volume loss after erosive challenge. Therefore, further studies are needed in human teeth to validate these findings and determine the optimal parameters of irradiation.

## Conclusions

The Er:YAG laser showed the lowest volume loss from wear analysis, suggesting the increased the acid resistance of dentin.
